# Synthesis, crystal structure and Hirshfeld surface analysis of *tert*-butyl *N*-acetyl­carbamate

**DOI:** 10.1107/S2056989022009483

**Published:** 2022-09-30

**Authors:** Aly Dawa El Mestehdi, Moctar Abba, Mohamed Lemine El Housseine, Abderrahmane Ould Hadou, Aliou Hamady Barry, Brahim Ould Elemine, Christian Jelsch, Mohamed Gaye

**Affiliations:** a Unité de Chimie Moléculaire et Environnement, Département de Chimie, FST, UNA, Nouakchott, Mauritania; b Département des Sciences Exactes, Ecole Normale Supérieure de Nouakchott, Nouakchott, Mauritania; c Agence Nationale de Recherches Géologiques et du Patrimoine Minier (ANARPAM), Nouakchott, Mauritania; d Laboratoire CRM^2^, CNRS, Institut Jean Barriol, Université de, Lorraine, 54000, Nancy, France; e Département de Chimie, Faculté des Sciences et Techniques, Université Cheik Anta Diop, Dakar, Senegal; University of Durham, England

**Keywords:** X-ray crystal structure, *tert*-butyl acetyl­carbamate, natural phosphate, Mauritanian phosphate deposit

## Abstract

This article reports a practical synthesis of *tert*-butyl acetyl­carbamate, C_7_H_13_NO_3_, from *N*-Boc-thio­acetamide and the study of its crystal structure. The reaction proceeds in the presence of natural phosphate as a catalyst, with excellent yield, simple workup and benign environment. The crystal structure was refined using a transferred multipolar atom model. In the crystal, symmetrical pairs of strong N—H⋯O hydrogen bonds connect the mol­ecules into dimers with an *R* ^2^_2_(8) ring motif. The inter­actions between neighbouring dimers are mostly van der Waals, between hydro­phobic methyl groups. Hirshfeld surface analysis shows the major contributions to the crystal packing are from H⋯H (42.6%) and O⋯H (26.7%) contacts.

## Abstract 2

The title compound, *tert*-butyl acetyl­carbamate, was synthesized by a green method using natural phosphate as a catalyst. The crystal packing shows pairs of mol­ecules forming dimers. These pairs generate centrosymmetric rings with 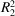 (8) motifs linked by a double N—H⋯O=C hydrogen bond. Hirshfeld surface analysis indicates that the major contributions to the crystal packing are from H⋯H (42.6%) and O⋯H (26.7%) contacts.

## 1. Chemical context

Carbamates are widely used as agrochemicals, in the polymer industry, in peptide synthesis (Dibenedetto *et al.*, 2002 ▸) and in medicinal chemistry, where many derivatives are specifically designed to make drug–target inter­actions through their carbamate moiety (Ghosh & Brindisi, 2015 ▸). Here we report the crystal structure of *tert*-butyl-acetyl­carbamate, C_7_H_13_NO_3_ (I), which we obtained while attempting to synthesize polyfunctional amidines (which are useful in synthetic fields, especially as templates for the development of various novel heterocycles) using heterogeneous catalysis on natural phosphates (NP) – readily available, stable, easy to handle and regenerate, non-toxic and inexpensive catalysts with both basic and acidic active sites (Sebti *et al.*, 1994 ▸, 1996 ▸). 
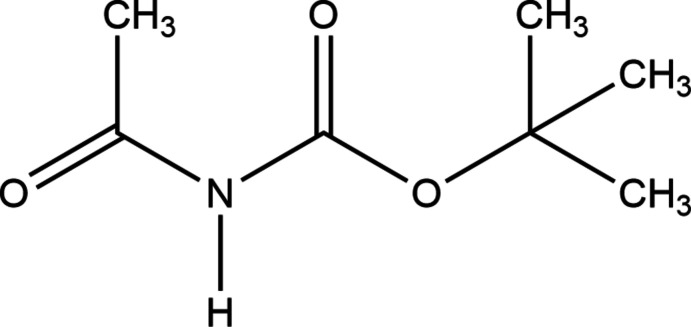


We followed the procedure described by Lee *et al.* (1998 ▸), but using natural phosphate (NP) as a catalyst instead of Lewis acids such as ZnCl_2_, Et_3_O^+^BF_4_^−^ and FeCl_2_. The synthesis was carried out by blending *N*-(t-Boc)thio­acetamide with various amino­esters, in the presence of NEt_3_ and NP. The reaction yielded (I) instead of the desired amidine, *i.e.* the sulfur atom was substituted by oxygen. In the absence of NP, no product was obtained and the starting materials were recovered.

## 2. Structural commentary

**Figure 1:**
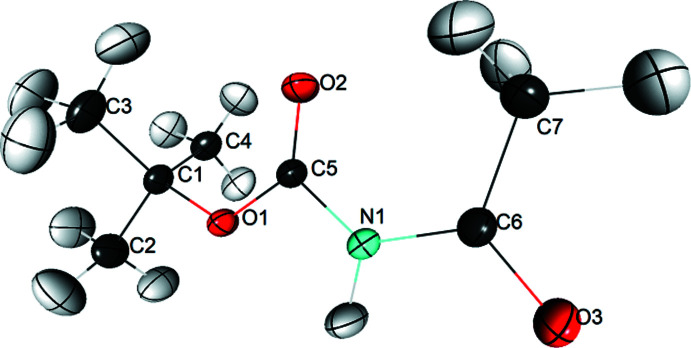
View of mol­ecule (I), showing the atom-labelling scheme. Displacement ellipsoids are drawn at the 50% probability level.

The title compound, C_7_H_13_NO_3_, (Fig. 1 ▸) crystallizes in the space group *P*2_1_/*n* with one mol­ecule per asymmetric unit. The skeleton of the mol­ecule is nearly planar if the C3 and C4 atoms are excluded, the root-mean-square deviation from the mean plane being 0.070 Å. The C3H_3_ and C4H_3_ methyl groups, located on either side of the mean plane, generate two weak intra­molecular hydrogen bonds with the carbonyl O2 atom located in the plane [C3—H3*C*⋯O2 and C4—H4*A*⋯O2, *d*(H⋯O)= 2.49 and 2.48 Å, respectively; Table 1 ▸].

## 3. Supra­molecular features

**Figure 2:**
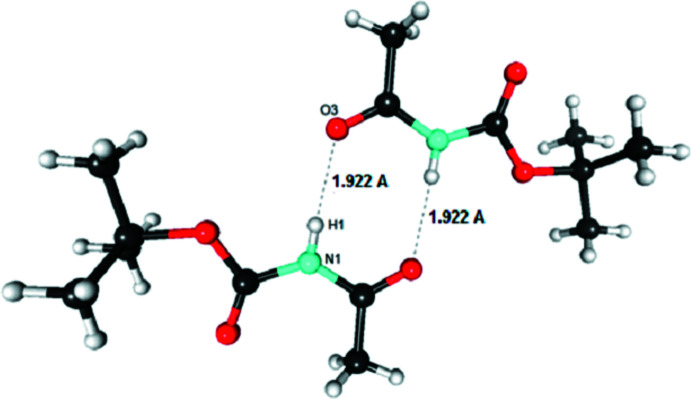
View of the mol­ecular dimer linked by a double hydrogen bond.

**Figure 3:**
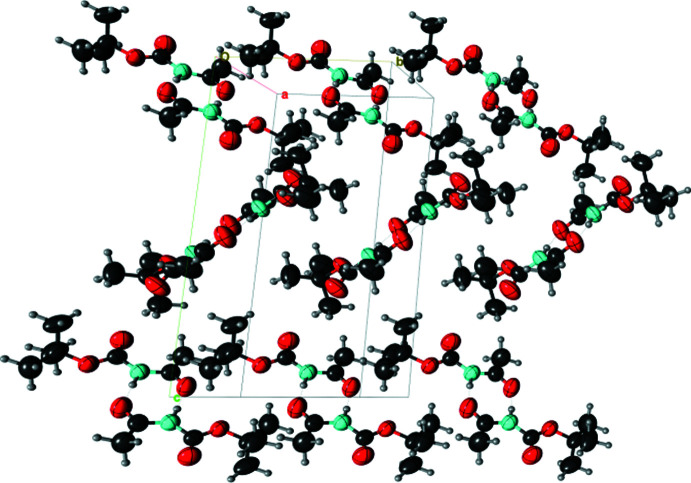
Mol­ecular packing of (I), viewed along the *a* axis, showing different orientations of the dimers.

In the crystal, a centrosymmetric dimer of mol­ecules is held together by two N—H⋯O=C hydrogen bonds, N1—H1⋯O3 and its symmetry equivalent [*d*(H⋯O) = 1.92 (1) Å, Table 1 ▸], which represent the strongest inter­actions in the packing and create an inversion-symmetric supra­molecular motif of graph-set 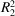 (8) (Fig. 2 ▸). Fig. 3 ▸ shows the packing of these dimers. If we consider the Hirshfeld surface around the dimer *as* a whole, this surface is constituted mainly by hydro­phobic (C and H-c) atoms (81%) and oxygen atoms (13%). The inter­actions between neighbouring dimers are mostly hydro­phobic: H-c⋯H-c between methyl groups (43%) and C⋯H-c between carbonyl and methyl groups (21%). Weak C—H⋯O hydrogen bonds also occur between dimers (23%). The steric hindrance of the methyl groups causes an offset of the mol­ecules of consecutive dimers, so that no strong hydrogen bond is observed between the dimers. Consequently, the crystal appears to be stabilized by strong hydrogen bonding within the dimers and van der Waals forces without.

## 4. Hirshfeld analysis

**Figure 4:**
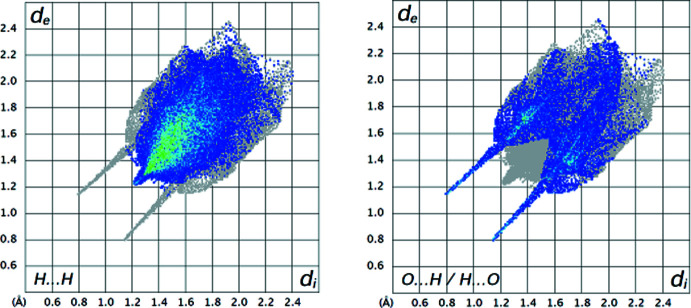
Two-dimensional fingerprint plots of the major contacts on the Hirshfeld surface.

**Figure 5:**
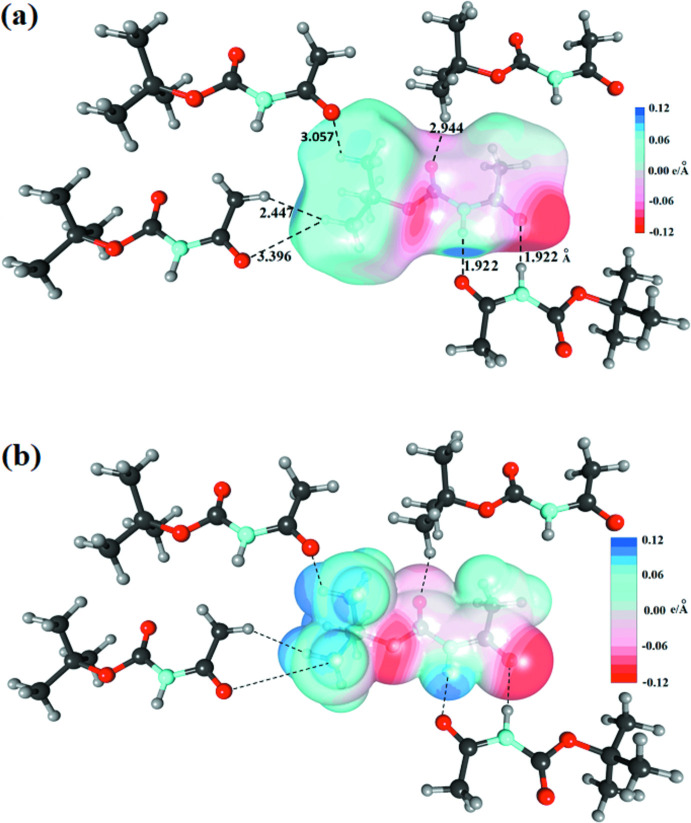
(*a*) Hirshfeld and (*b*) van der Waals surfaces around mol­ecule (I). The N—H⋯O and C—H⋯O hydrogen bonds as well as a short H⋯H contacts are shown. The surfaces are coloured according to the electrostatic potential.

*MoProViewer* (Jelsch *et al.*, 2005 ▸) was used to further investigate and visualize the inter­molecular inter­actions in the crystal. The Hirshfeld surface was computed from the model after multipolar refinement but using electron density from the spherical-neutral atom model. The 2D fingerprint plots (Fig. 4 ▸) were generated with *Crystal Explorer* (Spackman *et al.*, 2021 ▸). The most significant contributions for the contacts in the crystal packing (Table 2 ▸) are from H⋯H (46.2%), O⋯H/H⋯O (26.7%) and C⋯H/H⋯C contacts (18.7%), whereas only 2.8% are from N⋯H/H⋯N contacts. In the fingerprint plots (Fig. 4 ▸), the two reciprocal spikes at a short distance correspond to the O⋯H—N/N—H⋯O contacts, *i.e*. strong hydrogen bonds. The H⋯H contacts show also a small spike on the diagonal line, the shortest distances being 2.447 Å between H2*B* and H7*A*(*x* + 1, *y* − 1, *z*) (Fig. 5 ▸ *a*). The inter­molecular inter­actions were further evaluated by computing the enrichment ratios (*E*, see Table 2 ▸) in order to highlight which contacts are over-represented and are likely to represent energetically strong inter­actions and be the driving force in crystal formation (Jelsch *et al.*, 2014 ▸). The enrichment values are obtained as the ratio between the shares of actual contacts *C*_xy_ and the random (equiprobable) contacts *R*_xy_, the latter calculated as if all types of contacts had the same propensity to occur and are obtained by probability products (*R*_xy_ = *S*_x_·*S*_y_). The H-c⋯H-c hydro­phobic contacts are the most abundant on the Hirshfeld surface but have a unitary enrichment ratio. The O⋯H-c and C⋯H-c weak hydrogen bonds are the next most abundant inter­actions and are slightly enriched (*E* = 1.04 and 1.12, respectively). While the strong O⋯H-n hydrogen bonds in the fourth position represent only 5.9% of the contact surface, they are the most enriched at *E* = 3.41. The H-c⋯N contacts are over-represented with *E* = 1.46 as the nitro­gen atom inter­acts mostly with methyl groups on both sides of the *sp* ^2^ plane.

The Hirshfeld surface was partitioned into (H-c, C) and (H-n, O, N) atoms’ shares in order to analyse the contacts in terms of hydro­phobic and hydro­philic inter­actions. Overall, hydro­phobic atoms (C and H-c) comprise 77.5% of the surface, but the hydro­phobic contacts between these atoms (61.8%) are not significantly enriched at *E* = 1.03. Contacts between hydro­philic atoms (22.5% of the surface), mostly in the form of strong hydrogen bonds, are enriched to 6.7% (*E* = 1.32) while cross-inter­actions (between hydro­phobic and hydro­philic atoms) are under-represented (31.6%, *E* = 0.90).

The electrostatic potential was computed on the Hirshfeld and van der Waals surfaces of the mol­ecule (Fig. 5 ▸). The two surfaces show similar potential values which are both in the −0.12 to +0.12 e Å^−1^ range. The regions around the three oxygen atoms are electronegative while the NH group displays positive potential on the surface, followed by the methyl groups which are moderately electropositive.

## 5. Database survey

**Figure 6:**
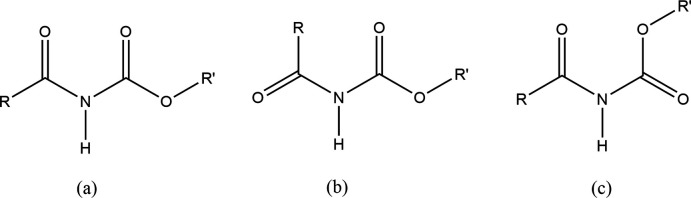
Conformations of amide-ester derivatives.

The Cambridge Structural Database (Version 5.43, November 2021 ▸; Groom *et al.*, 2016) was surveyed using *ConQuest* (version 2020.2.0; Bruno *et al.*, 2002 ▸). The eight-membered supra­molecular motif, with a double N—H⋯O=C hydrogen bond between two amide groups, is quite common, being encountered in 10,336 crystal structures. The amide-ester fragment, encountered in 35 structures, exists in three different near-planar conformations (Fig. 6 ▸). Conformation (*a*) with the *syn* disposition of C=O bonds appears in 23 structures, including the nearest reported analogue of (I), 1,1-di­methyl­ethyl-*N*-propano­ylcarbamate (II) (Brodesser *et al.*, 2003 ▸). Two different *anti* conformations, (*b*) and (*c*), are adopted by nine and three compounds, respectively. Mol­ecule (I) adopts the *anti* conformation (*b*). Compound (I) is the homologue of (II).

## 6. Synthesis and crystallization

*Materials and physical methods*. All reagents were purchased from Sigma-Aldrich. Reaction progress was monitored by thin-layer chromatography (TLC) on silica-gel plates (Fluka Kieselgel 60 F254). Flash chromatography purifications were performed on Inter­chim Puriflash (Puriflash columns 50 µ). X-ray fluorescence analysis was performed on a PANalytical AxiosmAX spectrometer.

*Preparation of the catalyst*. The NP used in this work comes from the Bofal phosphate deposit in Mauritania. Before being used in catalysis, it underwent quartering treatment, particle-size separation, aqueous dissolution, filtration and evaporation of water, calcination at 1173 K for 1h and grinding. The fraction of 60–100 µm grain size was used. The nominal chemical compositions of this phosphate were given by X-ray fluorescence (XRF) analysis. The total amount of the natural inorganic components was 90.86% (Table 3 ▸). The rest was mainly organic matter, as indicated by the weight loss on combustion, which amounted to 10.43%.

*Preparation of tert-butyl acetyl­carbamate* (*I).* It should be noted that compound (I) was prepared in our attempt to synthesize polyfunctional amidines, which are useful in synthetic fields, especially as a template for the development of various new heterocycles. In the preparation, we used the same operating conditions as Lee *et al.* (1998 ▸), substituting NP as the catalyst for a Lewis acid. To a solution of *N*-(t-Boc)thio­acetamide (87.6 mg; 0.5 mmol), the hydro­chloride salt of an amino ester (0.5 mmol) and tri­ethyl­amine (1.65 mmol) in a dry solvent (10 mL), NP (87.6 mg) was added with stirring. The reaction was stirred for 30 min at room temperature. The mixture was filtered through a pad of celite. The residue was purified by Inter­chim Puriflash (Puriflash columns 50 µ) using a cyclo­hexa­ne/ethyl acetate eluent system, to yield crystalline (I) in a very high yield (≥ 95%). We have tested this reaction with various solvents (THF, CH_3_CN and DMF) and hydro­chlorides of different amino esters, *viz*. glycine ethyl ester, l-valine methyl ester, l-alanine ethyl ester and l-phenyl­alanine methyl ester. *N*-(Boc)thio­acetamide was prepared as described in the literature (Lee *et al.*, 1998 ▸).

## 7. Refinement

Crystal data, data collection and structure refinement details are summarized in Table 4 ▸. A least-squares refinement, based on |*F*|^2^ of all reflections, was carried out with the program *MoPro* (Jelsch *et al.*, 2005 ▸) using the *ELMAM2* electron-density database (Domagała *et al.*, 2012 ▸). In this approach, scale factors, atomic positions and displacement parameters for all atoms were varied, but a multipolar charged-atom model was applied until convergence. The H—*X* distances were constrained to the standard values in neutron diffraction studies (Allen & Bruno, 2010 ▸). The anisotropic displacement parameters of hydrogen atoms were constrained to the values obtained from the *SHADE3* server (Madsen & Hoser, 2014 ▸). Two subsets of the mol­ecule (*O*-*t*-butyl moiety and the rest of the mol­ecule) were used as input to the *SHADE3* program to obtain better estimations of the *U*_ani_(H) displacement parameters. The use of a transferred multipolar atom model allowed the reduction of *R*(*F*) to 4.6% and *wR*_2_(*F* ^2^) to 7.2%, compared to 6.1% and 11.8%, respectively, for the neutral-spherical atom model, as refined in *MoPro*. The r.m.s. residual electron density was likewise reduced from 0.042 to 0.034 e Å^−3^.

## Supplementary Material

Crystal structure: contains datablock(s) I. DOI: 10.1107/S2056989022009483/zv2015sup1.cif

The hkl file is provided as suppemental material. DOI: 10.1107/S2056989022009483/zv2015sup3.txt

Supporting information file. DOI: 10.1107/S2056989022009483/zv2015Isup3.cml

CCDC reference: 2209649

Additional supporting information:  crystallographic information; 3D view; checkCIF report

## Figures and Tables

**Table 1:** Hydrogen-bond geometry (Å, °)

*D*—H⋯*A*	*D*—H	H⋯*A*	*D*⋯*A*	*D*—H⋯*A*
C4—H4*A*⋯O2	1.10	2.48	3.0651 (14)	112
C3—H3*C*⋯O2	1.10	2.49	2.9928 (15)	107
N1—H1⋯O3^i^	1.01 (1)	1.92 (1)	2.9285 (11)	173 (1)

Symmetry code: (i) 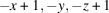 .

**Table 2:** Statistical analysis of inter­molecular contacts on the Hirshfeld surface H-c and H-n signify hydrogen atoms bound to C (hydro­phobic) and N (hydro­philic), respectively. Reciprocal contacts (*X*⋯*Y* and *Y*⋯*X*) are merged. The most prevalent and enriched contacts are highlighted in bold.

Atom	H-n	N	O	H-c	C
*S*_*x*_ (%)	5.5	1.5	15.5	64.0	13.5
*C_xy_* (%) (*E*_xy_)					
H-n	0.4 (1.39)				
N	0 (0)	0 (0)			
O	5.9 (**3.41**)	0 (0)	0.4 (0.16)		
H-c	4.2 (0.59)	2.8 (**1.46**)	**20.8** (1.04)	**41.6** (0.99)	
C	0.2 (0.13)	0.1 (0.41)	3.4 (0.86)	**18.5** (**1.12**)	1.6 (1.00)

**Table 3:** X-ray fluorescence (XRF) analysis (%) of natural phosphate

SiO_2_	TiO_2_	Al_2_O_3_	Fe_2_O_3_	MgO	CaO	Na_2_O	K_2_O	MnO	P_2_O_5_	SO_3_
14.17	0.058	17.51	0.530	0.245	31.66	0.319	0.113	0.016	26.18	0.060

**Table 4:** Experimental details

Crystal data	
Chemical formula	C_7_H_13_NO_3_
*M*_r_	159.18
Crystal system, space group	Monoclinic, *P*2_1_/*n*
Temperature (K)	293
*a*, *b*, *c* (Å)	6.0404 (6), 8.6114 (7), 17.6110 (17)
β (°)	98.771 (9)
*V* (Å^3^)	905.35 (15)
*Z*	4
Radiation type	Mo *K*α
μ (mm^−1^)	0.09
Crystal size (mm)	0.15 × 0.1 × 0.08

Data collection	
Diffractometer	Bruker Kappa CCD
Absorption correction	–
No. of measured, independent and observed [*I* > 2 σ(*I*)] reflections	2405, 2059, 1627
*R*_int_	0.035
(sin θ/λ)_max_ (Å^−1^)	0.650

Refinement	
*R*[*F* ^2^ > 2σ(*F* ^2^)], *wR*(*F* ^2^), *S*	0.034, 0.072, 1.00
No. of reflections	2059
No. of parameters	139
No. of restraints	31
H-atom treatment	Only H-atom coordinates refined
Δρ_max_, Δρ_min_ (e Å^−3^)	0.16, −0.17

Computer programs: *APEX3* and *SAINT* (Bruker, 2016 ▸), *SHELXS97* (Sheldrick, 2008 ▸) and *MoPro* (Jelsch *et al.*, 2005 ▸).

## supplementary crystallographic information

### Crystal data

**Table:**

C_7_H_13_NO_3_	*F*(000) = 344
*M**_r_* = 159.18	*D*_x_ = 1.168 Mg m^−^^3^
Monoclinic, *P*2_1_/*n*	Mo *K*α radiation, λ = 0.71073 Å
Hall symbol: -P 2yn	Cell parameters from 4200 reflections
*a* = 6.0404 (6) Å	θ = 2.4–28.6°
*b* = 8.6114 (7) Å	µ = 0.09 mm^−^^1^
*c* = 17.6110 (17) Å	*T* = 293 K
β = 98.771 (9)°	Block, colorless
*V* = 905.35 (15) Å^3^	0.15 × 0.1 × 0.08 mm
*Z* = 4	

### Data collection

**Table:**

Bruker Kappa CCD diffractometer	1627 reflections with *I* > 2 σ(*I*)
Radiation source: fine-focus sealed tube	*R*_int_ = 0.035
Graphite monochromator	θ_max_ = 27.5°, θ_min_ = 2.6°
CCD scans	*h* = −7→7
2405 measured reflections	*k* = 0→11
2059 independent reflections	*l* = 0→22

### Refinement

**Table:**

Refinement on *F*^2^	Secondary atom site location: difference Fourier map
Least-squares matrix: full	Hydrogen site location: difference Fourier map
*R*[*F*^2^ > 2σ(*F*^2^)] = 0.034	Only H-atom coordinates refined
*wR*(*F*^2^) = 0.072	*w* = 1/[4.4*σ^2^(*F*_o_^2^)]
*S* = 1.00	(Δ/σ)_max_ = 0.002
2059 reflections	Δρ_max_ = 0.16 e Å^−^^3^
139 parameters	Δρ_min_ = −0.17 e Å^−^^3^
31 restraints	Extinction correction: Isotropic Gaussian
Primary atom site location: structure-invariant direct methods	Extinction coefficient: 0.51132

### Special details

**Table:**

Refinement. Refinement of F^2^ against reflections. The threshold expression of F^2^ > 2sigma(F^2^) is used for calculating R-factors(gt) and is not relevant to the choice of reflections for refinement. R-factors based on F^2^ are statistically about twice as large as those based on F, and R-factors based on ALL data will be even larger.

### Fractional atomic coordinates and isotropic or equivalent isotropic displacement parameters (Å^2^)

**Table:**

	*x*	*y*	*z*	*U*_iso_*/*U*_eq_	
O1	0.38211 (11)	0.355396 (15)	0.40123 (4)	0.05484 (14)	
O2	0.63464 (12)	0.29985 (8)	0.32233 (4)	0.06801 (17)	
O3	0.73388 (12)	−0.08048 (8)	0.46784 (4)	0.06574 (16)	
N1	0.57479 (13)	0.14069 (8)	0.42246 (5)	0.05046 (16)	
H1	0.474 (2)	0.1271 (16)	0.4629 (7)	0.08754	
C5	0.54053 (15)	0.27070 (10)	0.37571 (5)	0.04754 (17)	
C6	0.73373 (15)	0.02549 (11)	0.42216 (6)	0.05121 (18)	
C1	0.30097 (15)	0.50254 (10)	0.36327 (6)	0.05446 (18)	
C7	0.89889 (19)	0.03248 (14)	0.36770 (7)	0.0716 (3)	
H7A	1.0210 (16)	−0.0607 (14)	0.3829 (8)	0.10454	
H7B	0.9899 (18)	0.1427 (14)	0.3755 (9)	0.11089	
H7C	0.818 (2)	0.0353 (17)	0.3076 (6)	0.11503	
C4	0.18467 (19)	0.46831 (14)	0.28329 (7)	0.0740 (3)	
H4A	0.3039 (17)	0.4270 (16)	0.2468 (6)	0.10880	
H4B	0.106 (2)	0.5761 (14)	0.2597 (8)	0.11611	
H4C	0.0505 (16)	0.3836 (15)	0.2852 (8)	0.11411	
C3	0.4904 (2)	0.61607 (13)	0.36559 (8)	0.0758 (2)	
H3A	0.578 (2)	0.6274 (16)	0.4245 (8)	0.11708	
H3B	0.419 (2)	0.7310 (12)	0.3505 (9)	0.12094	
H3C	0.6142 (17)	0.5881 (15)	0.3283 (7)	0.10779	
C2	0.1345 (2)	0.55587 (15)	0.41419 (8)	0.0822 (3)	
H2A	0.0073 (16)	0.4648 (14)	0.4127 (9)	0.11714	
H2B	0.054 (2)	0.6620 (14)	0.3891 (8)	0.12347	
H2C	0.232 (2)	0.5828 (17)	0.4703 (7)	0.12492	

### Atomic displacement parameters (Å^2^)

**Table:**

	*U*^11^	*U*^22^	*U*^33^	*U*^12^	*U*^13^	*U*^23^
O1	0.0660 (4)	0.0489 (4)	0.0515 (4)	0.0085 (3)	0.0150 (3)	0.0127 (3)
O2	0.0786 (5)	0.0669 (5)	0.0644 (5)	0.0061 (4)	0.0300 (4)	0.0184 (3)
O3	0.0745 (5)	0.0484 (4)	0.0771 (5)	0.0077 (3)	0.0204 (4)	0.0162 (3)
N1	0.0599 (5)	0.0439 (4)	0.0495 (5)	0.0021 (3)	0.0144 (3)	0.0091 (3)
H1	0.09872	0.08344	0.09180	0.02741	0.05087	0.03885
C5	0.0539 (5)	0.0441 (5)	0.0456 (5)	−0.0003 (4)	0.0107 (4)	0.0056 (4)
C6	0.0573 (5)	0.0430 (5)	0.0533 (5)	−0.0001 (4)	0.0083 (4)	0.0024 (4)
C1	0.0592 (5)	0.0460 (5)	0.0551 (5)	0.0050 (4)	−0.0011 (4)	0.0103 (4)
C7	0.0720 (7)	0.0747 (7)	0.0731 (7)	0.0168 (6)	0.0274 (6)	0.0133 (6)
H7A	0.09887	0.10412	0.11797	0.04100	0.04001	0.02395
H7B	0.08822	0.09468	0.16018	−0.00028	0.05229	0.03275
H7C	0.14423	0.13621	0.06953	0.04903	0.03193	0.00590
C4	0.0757 (7)	0.0794 (8)	0.0597 (7)	−0.0079 (6)	−0.0129 (5)	0.0126 (6)
H4A	0.12776	0.13382	0.06263	0.00285	0.00747	0.00354
H4B	0.11933	0.11272	0.10174	0.01287	−0.02973	0.03707
H4C	0.09893	0.11674	0.11538	−0.03490	−0.01984	0.00269
C3	0.0723 (7)	0.0514 (6)	0.0968 (9)	−0.0083 (5)	−0.0096 (6)	0.0110 (6)
H3A	0.11467	0.09511	0.12396	−0.01659	−0.03780	−0.00724
H3B	0.11702	0.05611	0.17940	−0.00332	−0.01031	0.02819
H3C	0.08755	0.09430	0.14362	−0.01523	0.02433	0.02842
C2	0.0844 (8)	0.0751 (8)	0.0889 (9)	0.0249 (6)	0.0193 (6)	0.0046 (7)
H2A	0.09735	0.11323	0.15160	0.01562	0.05342	0.02003
H2B	0.12561	0.09416	0.15181	0.05340	0.02495	0.01964
H2C	0.15392	0.13217	0.08898	0.04922	0.01950	−0.01800

### Geometric parameters (Å, º)

**Table:**

O1—C5	1.3344 (11)	C7—H7A	1.0950
O1—C1	1.4808 (9)	C7—H7B	1.0950
O2—C5	1.1970 (11)	C4—H4C	1.0950
O3—C6	1.2164 (11)	C4—H4A	1.0950
N1—C5	1.3864 (12)	C4—H4B	1.0950
N1—C6	1.3811 (12)	C3—H3C	1.0950
N1—H1	1.013 (8)	C3—H3B	1.0950
C6—C7	1.4868 (15)	C3—H3A	1.0950
C1—C3	1.5009 (14)	C2—H2C	1.0950
C1—C4	1.5042 (15)	C2—H2B	1.0950
C1—C2	1.5170 (16)	C2—H2A	1.0950
C7—H7C	1.0950		
			
C5—O1—C1	121.38 (6)	H7C—C7—H7B	104 (1)
C5—N1—C6	128.22 (7)	H7A—C7—H7B	107.3 (8)
C5—N1—H1	117.3 (7)	C1—C4—H4C	110.1 (7)
C6—N1—H1	114.4 (7)	C1—C4—H4A	111.0 (6)
O1—C5—O2	126.91 (8)	C1—C4—H4B	107.6 (6)
O1—C5—N1	106.93 (7)	H4C—C4—H4A	111 (1)
O2—C5—N1	126.15 (7)	H4C—C4—H4B	107.3 (10)
O3—C6—N1	117.82 (7)	H4A—C4—H4B	110 (1)
O3—C6—C7	121.61 (8)	C1—C3—H3C	115.4 (7)
N1—C6—C7	120.57 (8)	C1—C3—H3B	108.2 (6)
O1—C1—C3	110.28 (7)	C1—C3—H3A	109.8 (7)
O1—C1—C4	109.28 (7)	H3C—C3—H3B	109 (1)
O1—C1—C2	101.29 (7)	H3C—C3—H3A	108.3 (10)
C3—C1—C4	113.48 (8)	H3B—C3—H3A	105 (1)
C3—C1—C2	110.99 (9)	C1—C2—H2C	106.5 (7)
C4—C1—C2	110.85 (9)	C1—C2—H2B	107.8 (8)
C6—C7—H7C	112.4 (7)	C1—C2—H2A	107.1 (7)
C6—C7—H7A	107.8 (6)	H2C—C2—H2B	109.3 (10)
C6—C7—H7B	109.1 (7)	H2C—C2—H2A	117 (1)
H7C—C7—H7A	115.8 (9)	H2B—C2—H2A	108.8 (10)
			
O1—C5—N1—C6	−174.66 (12)	H1—N1—C6—C7	−178 (1)
O1—C5—N1—H1	4 (1)	C5—O1—C1—C3	−60.18 (11)
O1—C1—C3—H3C	71.1 (8)	C5—O1—C1—C4	65.21 (11)
O1—C1—C3—H3B	−166.1 (9)	C5—O1—C1—C2	−177.77 (12)
O1—C1—C3—H3A	−51.6 (9)	C5—N1—C6—C7	1.01 (14)
O1—C1—C4—H4C	55.5 (7)	C4—C1—C3—H3C	−51.9 (7)
O1—C1—C4—H4A	−67.8 (7)	C4—C1—C3—H3B	70.9 (7)
O1—C1—C4—H4B	172.1 (9)	C4—C1—C3—H3A	−174.6 (9)
O1—C1—C2—H2C	66.4 (7)	C4—C1—C2—H2C	−177.8 (9)
O1—C1—C2—H2B	−176.4 (7)	C4—C1—C2—H2B	−60.5 (7)
O1—C1—C2—H2A	−59.4 (7)	C4—C1—C2—H2A	56.4 (8)
O2—C5—O1—C1	0.45 (12)	H4A—C4—C1—C3	56 (1)
O2—C5—N1—C6	5.90 (14)	H4A—C4—C1—C2	−179 (1)
O2—C5—N1—H1	−175 (1)	H4B—C4—C1—C3	−64 (1)
O3—C6—N1—C5	−179.23 (13)	H4B—C4—C1—C2	61 (1)
O3—C6—N1—H1	2 (1)	H4C—C4—C1—C3	179 (1)
O3—C6—C7—H7C	121.4 (9)	H4C—C4—C1—C2	−55 (1)
O3—C6—C7—H7A	−7.4 (8)	C3—C1—C2—H2C	−50.7 (7)
O3—C6—C7—H7B	−123.6 (8)	C3—C1—C2—H2B	66.5 (8)
N1—C5—O1—C1	−178.99 (12)	C3—C1—C2—H2A	−176.5 (8)
N1—C6—C7—H7C	−58.8 (8)	H3A—C3—C1—C2	60 (1)
N1—C6—C7—H7A	172.4 (8)	H3B—C3—C1—C2	−55 (1)
N1—C6—C7—H7B	56.1 (8)	H3C—C3—C1—C2	−177 (1)

### Hydrogen-bond geometry (Å, º)

**Table:**

*D*—H···*A*	*D*—H	H···*A*	*D*···*A*	*D*—H···*A*
C4—H4*A*···O2	1.10	2.48	3.0651 (14)	112
C3—H3*C*···O2	1.10	2.49	2.9928 (15)	107
N1—H1···O3^i^	1.01 (1)	1.92 (1)	2.9285 (11)	173 (1)

Symmetry code: (i) −*x*+1, −*y*, −*z*+1.
